# Spina Ventosa

**Published:** 1891-01-10

**Authors:** 


### SPINA VENTOSA.

Before the tubercular nature of this affection was demonstrated by Volkmann, its treatment was most unsatisfactory, and in our text-books we read of perfect-rest, splints, counter-irritants, &c. Dr. Unger, reporting on cases treated by him at the Jewish hospital in Berlin, discusses the relative frequency with which the several bones are affected and the mode of treatment, which should always be by operation for the extirpation of the tubercular forms. Of his 80 cases, 45 were under five years of age, 30 between five and ten, and five between ten and fifteen. The bones of the metacarpus were the seat of the disease in 40 instances, and the phalanges of the fingers in 38. The metatarsus in 13, and the phalanges of the toes in 3. A like condition was met with in the ulna in 3, the radius in 2, the tibia in 3, and the lower jaw in 2. Early operation is imperative, but the nature of the procedure must be determined by the extent and stage of the disease. 1. Scooping out with the sharp spoon suffices so long as the diaphysis or shaft of the bone is alone diseased. 2. Excision of the entire bone if the epiphysis be involved. 3. Amputation of the limb or member if the disease be so far advanced as to preclude all hope of its use being restored, or if life be endangered by further extension or by exhaustion.

